# Performance of DNA metabarcoding, standard barcoding, and morphological approach in the identification of host–parasitoid interactions

**DOI:** 10.1371/journal.pone.0187803

**Published:** 2017-12-13

**Authors:** Martin Šigut, Martin Kostovčík, Hana Šigutová, Jiří Hulcr, Pavel Drozd, Jan Hrček

**Affiliations:** 1 Department of Biology and Ecology/Institute of Environmental Technologies, University of Ostrava, Ostrava, Czech Republic; 2 Department of Genetics and Microbiology, Charles University in Prague, Praha, Czech Republic; 3 BIOCEV, Institute of Microbiology, Academy of Sciences of the Czech Republic, Vestec, Czech Republic; 4 School of Forest Resources and Conservation, University of Florida-IFAS, Gainesville, Florida, United States of America; 5 Entomology and Nematology Department, University of Florida-IFAS, Gainesville, Florida, United States of America; 6 Biology Centre of the Czech Academy of Sciences, Institute of Entomology, České Budějovice, Czech Republic; University of Guelph, CANADA

## Abstract

Understanding interactions between herbivores and parasitoids is essential for successful biodiversity protection and monitoring and for biological pest control. Morphological identifications employ insect rearing and are complicated by insects’ high diversity and crypsis. DNA barcoding has been successfully used in studies of host–parasitoid interactions as it can substantially increase the recovered real host–parasitoid diversity distorted by overlooked species complexes, or by species with slight morphological differences. However, this approach does not allow the simultaneous detection and identification of host(s) and parasitoid(s). Recently, high-throughput sequencing has shown high potential for surveying ecological communities and trophic interactions. Using mock samples comprising insect larvae and their parasitoids, we tested the potential of DNA metabarcoding for identifying individuals involved in host–parasitoid interactions to different taxonomic levels, and compared it to standard DNA barcoding and morphological approaches. For DNA metabarcoding, we targeted the standard barcoding marker cytochrome oxidase subunit I using highly degenerate primers, 2*300 bp sequencing on a MiSeq platform, and RTAX classification using paired-end reads. Additionally, using a large host–parasitoid dataset from a Central European floodplain forest, we assess the completeness and usability of a local reference library by confronting the number of Barcoding Index Numbers obtained by standard barcoding with the number of morphotypes. Overall, metabarcoding recovery was high, identifying 92.8% of the taxa present in mock samples, and identification success within individual taxonomic levels did not significantly differ among metabarcoding, standard barcoding, and morphology. Based on the current local reference library, 39.4% parasitoid and 90.7% host taxa were identified to the species level. DNA barcoding estimated higher parasitoid diversity than morphotyping, especially in groups with high level of crypsis. This study suggests the potential of metabarcoding for effectively recovering host–parasitoid diversity, together with more accurate identifications obtained from building reliable and comprehensive reference libraries, especially for parasitoids.

## Introduction

Parasitoids constitute a major component of global insect diversity and play important ecological roles, such as influencing the population dynamics of their hosts, contributing to the ecological stability and biological diversity of terrestrial ecosystems [1, 2]. Tritrophic interactions involving plants, herbivores, and parasitoids are thus crucial for ecological and evolutionary processes such as specialization and species diversification [3, 4]. Monitoring of ecological networks can help to determine the impact of environmental changes on ecosystem, and is essential for successful habitat management and restoration [5, 6]. Fast and cheap monitoring of species diversity and trophic interactions is also crucial for sustaining the productivity of agroecosystems [7]. Unfortunately, reconstructions of the above mentioned tritrophic interactions are still complicated, mainly due to the enormous amount of biological material to be processed [8]. Moreover, research on parasitoids’ biology, host specificity, and species richness is itself challenging because of their diversity, concealed nature, and ephemeral occurrence of adults [9].

Previous studies on host–parasitoid interactions were primarily based on insect rearing in combination with dissections, which are technically demanding and time-consuming. Traditionally used morphological identification of parasitoids is very difficult due to their diversity, intraspecific morphological plasticity, and complex life cycles [2, 10, 11]. Furthermore, morphological approach fails to reveal cryptic species, which are frequently present and have extraordinary host specificity, therefore leading to an underestimation of trophic links [12]. Recently, DNA barcodes have been successfully employed in parasitoid studies as they can substantially increase the recovered real host–parasitoid diversity, often distorted by overlooked species complexes or by species with slight morphological differences [12–14]. However, standard DNA barcoding does not allow simultaneous detection and identification of both the host and the parasitoid(s) [15].

High-throughput sequencing (HTS) techniques such as metabarcoding have significantly affected the scale and the precision of the outcomes from ecological studies. In DNA metabarcoding, entire communities are simultaneously explored by combining PCR amplification of universal markers and HTS. This technique has already revolutionized microbiology and mycology due to the permanently increasing availability and throughput of sequencing technologies. In fact, these technologies are driving the evolution of entirely new research lines on metazoan diversity, such as metasystematics [16, 17]. Moreover, metabarcoding is a promising neat solution for investigating symbiotic microorganisms along with the presence of their insect hosts [18]. However, given its novelty, DNA metabarcoding protocols in animal diversity studies are subject of ongoing debate, and multiple alternative strategies have been proposed [19]. While the approach yields unprecedented volumes of biodiversity data, it also includes many potential biases that remain to be fully explored and/or addressed, including its reliance on only one or a few markers such as mitochondrial DNA [20], unequal PCR amplification of markers from different species (e.g. [21]), incomplete or poorly curated taxonomic reference libraries [19], and algorithms for clustering sequences into taxonomic units.

Recently, metabarcoding has been successfully used in the reconstruction of (plant)–insect–parasitoid interactions [5]. Nested metabarcoding was applied to resolve the extent of parasitism throughout a population of an invasive lepidopteran host at the individual level [6]. While the authors concentrated on the specific problem of a single host species and its known range of parasitoids, more accurate identifications are still needed for more generalized studies on food webs and host–parasitoid interactions. Therefore, to fully utilize available barcode databases, we tested an almost full-length cytochrome oxidase subunit I (COI) mtDNA barcode (2*300 bp) that was amplified using degenerate primers with wide coverage [22], and employed a RTAX classifier, which enabled exploiting both non-overlapping reads to increase taxonomic assignment accuracy and precision in the study of mock host–parasitoid communities.

In the present study, we propose using DNA metabarcoding as an alternative to the comparisons between morphological and standard barcoding approaches used in previous methodological studies of host–parasitoid interactions (e.g. [12, 13]). Pros and cons of each method are extensively discussed. Based on a dataset of parasitoids and their hosts from 0.2 ha of broadleaf deciduous forest in Central Europe (Czech Republic), we aimed to test the possible use of DNA metabarcoding for identifying host–parasitoid interactions and compare it to standard DNA barcoding and morphological identifications. For the comparison of these three methods, we used five mock samples consisting of host caterpillars and their parasitoids, which were prepared by mixing host remnants with parasitoids reared or dissected from their hosts. Moreover, using the whole dataset, we compared the accuracy of molecular (DNA barcoding) and morphological approaches for the potential reconstruction of a local food web. We compared the number of Barcoding Index Numbers (BINs; i.e. putative species) obtained by standard COI mtDNA barcoding to the number of morphotypes. Moreover, as the success of molecular identifications depends on comprehensive reference libraries, we assess the completeness and usability of a local reference library for individual host and parasitoid groups.

## Materials and methods

### Ethics statement

No specific permits were required for fieldwork, as the sampled locality is not protected. The sampled area is owned by the Židlochovice Forest Enterprise, a division of the Czech National Forests, Inc., and the data were collected with their approval. No specific permissions were required to collect insect specimens, because the collected taxa are not protected in the Czech Republic.

### Insect sampling and rearing

Lepidoptera and Hymenoptera (sawflies) larvae were sampled from all plants with a diameter at breast height (DBH) >5 cm on a 0.2 ha plot of broadleaf deciduous forest in Lanžhot, Czech Republic (48.689685 N, 16.944742 E), using an elevated truck-mounted work platform (cherry-picker). Sampling was conducted from May to August 2013 and 2014. Each larva was morphotyped and photographed. Larvae were then transferred to plastic containers (one larva per container) where they were reared on fresh leaf material from the plant species it was collected from (*Acer campestre*, *Carpinus betulus*, *Fraxinus angustifolia*, *F*. *excelsior*, *Quercus cerris*, *Q*. *robur*, *Tilia cordata*, or *Ulmus laevis*) in the laboratory, until either adults or parasitoids emerged [23]. In total, 6473 hosts were sampled and 1700 parasitoids were reared (from 1032 rearing events).

### Morphological, standard barcoding, and metabarcoding identifications

We prepared five host–parasitoid, multispecies, mock samples consisting of 22 individual insects (five hosts and 17 parasitoids) representing 14 BINs (S1 Table). Every sample was prepared by mixing the remnants of the host’s body with parasitoids reared or dissected from it. To increase the complexity of samples before DNA extraction, we added body parts from parasitoids reared from other host individuals into mock samples 1–4 (from the same host species, except of sample 4).

#### Morphological identification

Host remnants were identified by appropriate specialists (listed in acknowledgements). Specimens in poor condition were identified by a combination of larval stage photographs, morphotype assignment, and morphology of the adults reared from larvae of the same morphotype. Additionally, we dissected the remnants of host larvae under a binocular microscope and explored the presence of remaining parasitoid developmental stages [11, 13]. Reared or dissected parasitoids were identified by M. Šigut using taxonomic keys and online databases (S2 Table) or by comparing them with reference collections held at the Zoologische Staatssammlung, Munich, Germany (ZSM). Problematic specimens were identified in consultation with expert taxonomist (Stefan Schmidt—curator for Hymenoptera, ZSM).

#### Standard barcoding identification

Tissue samples were taken from each parasitized host and parasitoid developmental stage (reared or dissected) and used in Sanger sequencing. In hosts, DNA was extracted from a small amount of skin or head tissue, whereas in parasitoids it was extracted from a single leg, egg, larva, or pupa using a Nucleospin Tissue Kit (Macherey-Nagel), following the manufacturer’s instructions. We obtained standard 658-bp COI barcodes using the general insect primers LepF1/LepR1 [24], or combinations of the internal primers LepF1/C_ANTMR1D and MLepF1/LepR1 for parasitoids, and LepF1/MLepR1 and MLepF1/LepR1 for lepidopteran hosts [12, 14]. Each PCR had a total volume of 20 μl and contained 13.3 μl molecular biology grade water, 4 μl 5× MyTaq^™^ Red Reaction Buffer, 0.1 μl MyTaq^™^ Red DNA Polymerase (all Bioline), 0.8 μM each primer (Sigma-Aldrich), and 1 μl genomic DNA. The amplification profile was as follows: one cycle of 1 min at 94°C for initial denaturation; five cycles of 40 s at 94°C, 40 s at 45°C, and 1 min at 72°C; 35 cycles of 40 s at 94°C, 40 s at 51°C, and 1 min at 72°C; and a final extension step of 5 min at 72°C. The resulting PCR products were visualized on a 1.5% agarose gel and then bi-directionally sequenced using BigDye^®^ Terminator v.3.1 (Applied Biosystems) on an ABI 3730XL sequencer (Macrogen Inc., Seoul, South Korea). Forward and reverse sequences were assembled to contigs and aligned in Bioedit [25]. Sequences were identified using the BOLD-IDS tool (http://www.boldsystems.org/index.php/IDS_OpenIdEngine); when no identification was obtained, a neighbor joining (NJ) tree including the 99 most similar sequences plus the query sequence was constructed, and the reference sequence with the shortest distance and divergence up to 3% was considered as the specimen identification [26]. Sequences of all specimens were deposited in GenBank (https://www.ncbi.nlm.nih.gov/genbank/; Accessions KY421520–KY421541).

#### Metabarcoding identification

Five mock samples consisting of host and parasitoid body parts were subjected to sequencing library preparation. We performed DNA extractions from mixed tissues following the same protocol as for standard barcoding (see above). To ensure broad recovery of taxa from mock samples, we used highly-degenerate primers for the amplification of COI markers: Fol-degen-for 5’-TCNACNAAYCAYAARRAYATYGG-3’ and Fol-degen-rev 5’-TANACYTCNGGRTGNCCRAARAAYCA-3’ [22]. A two-step PCR design was applied in library preparation. For the purposes of the present study, i.e. testing the metabarcoding performance, first-step amplifications were performed in quintuple reactions using only degenerate gene-specific primers to avoid PCR artifacts, caused by long primers with attached sequencing adapters and identifiers, and account for the stochasticity of PCR amplification [27, 28]. Each reaction volume (25 μl) contained 12.25 μl molecular biology grade water, 5 μl Q5 high fidelity 5× buffer, 0.2 mM dNTPs, 0.8 μM each primer, 1.25 U Q5-high fidelity polymerase (all New England BioLabs, Inc.) and 20 ng template DNA (measured fluorometrically on Qubit^™^, Thermofisher Scientific). In this first-step PCR a touch-down cycling program was applied [22], with minor modifications, based on our prior PCR optimizations to obtain robust amplicon recovery, including mainly increase of denaturation time, higher initial annealing temperature, and decrease of elongation time: initial denaturation at 95°C for 5 min; 10 cycles at 95°C for 30 s, 55°C for 45 s with decreasing annealing temperature by 1°C every cycle, and 72°C for 90 s; 15 cycles at 95°C for 30 s, 45°C for 45 s, 72°C for 90 s; and a final extension at 72°C for 10 min. Primers were synthesized with the last two bases modified by phosphorothioate bonds to inhibit degradation by proofreading polymerase. To prevent potential depletion of certain primers from the degenerate mixture we minimized the number of cycles. First-step quintuple PCR reactions were pooled, purified (UltraClean^®^ PCR clean-up kit, MoBio), and subjected to a second-step PCR including 15 amplification cycles with fused primers containing degenerate gene-specific primers, different 7-bp multiplex identifiers (MIDs; or barcodes) on the forward primer only to identify each sample, and Illumina^™^ (Illumina, Inc.) adapters. This second-step PCR was set up as the previous one, using 2 μl of the first-step PCR products as templates. The cycling profile consisted of an initial denaturation at 95°C for 2 min followed by 15 cycles at 95°C for 15 s, 51°C for 30 s, and 72°C for 1 min, and a final extension at 72°C for 10 min. The resulting PCR products were purified and checked using an agarose gel, as described for the first-step PCR, and quantified with the Quant-iT kit (Life Technologies). Equimolar proportions of all samples were subsequently pooled to create a final sequencing library at 7.5 ng/μl, which was submitted to paired-end sequencing on a MiSeq instrument (Illumina) at the Interdisciplinary Center for Biotechnology Research, University of Florida, United States, producing 2*300 bp long reads. Samples from this study comprised 1/1000^th^ of the whole sequencing output. Raw demultiplexed sequencing data with sample annotations are available at the Short Read Archive (SRA) database (http://www.ncbi.nlm.nih.gov/Traces/sra/) under accession SRP045622, and further details can be found under the Bioproject accession PRJNA258490.

Sequencing data were processed using QIIME 1.8.0 [29] and implementing scripts for all partial raw data processing steps (http://qiime.org/scripts/index.html), including quality checking, demultiplexing, read clustering, and taxonomic assignments. Such procedures allowed obtaining a matrix of recovered taxa per individual mock sample. We processed forward and reverse reads separately as they did not overlap; nevertheless, for classification purposes, we used information from both directions as described below. We subjected all reads to quality filtering and demultiplexing with default settings, including a maximum unacceptable phred quality score of 20, maximum number of consecutive bad quality base calls of 3, and maximum of 1.5 errors in the barcode. Reads shorter than 100 bp were discarded. We detected and filtered chimeras by implementing the *de novo* chimera identification of the USEARCH algorithm [30]. We used UCLUST [31] with a 97% similarity threshold to cluster filtered reads into molecular operational taxonomic units (MOTUs). Before producing the final dataset, we discarded singletons (clusters with only one read in individual samples). We subsequently classified representative sequences from all clusters implementing the RTAX method [32], which was developed for increasing the precision of assignment based on both reads from paired-end non-overlapping datasets against the standard reference COI database at iBOL [26] containing 150,610 COI barcode sequences (http://www.barcodinglife.org/index.php/datarelease, release 4.75—v1 on March 31, 2014). This method essentially provides taxonomic assignment based on a consensus algorithm. Reads from forward and reverse datasets were concurrently queried against the database, and only reference sequences that matched both paired reads were retained to calculate average percentage identity. Finally, only matches with an average identity within 0.5% of the maximum observed for that query sequence were retained and taxonomic assignment was based on the rank matching at least 50% of the hits. This threshold value was selected based on the preliminary tests run by the authors of the algorithm [32]. Utilizing both paired-end reads in parallel is expected to significantly enhance the classification’s rate, accuracy, and precision. Finally, we compiled information on read counts for all MOTU clusters from all samples together with taxonomic information into a MOTU table. We evaluated two factors of usability for this metabarcoding approach: recovery of diversity and ability to provide identifications up to the species level. We queried sequences that had no suitable match in the iBOL database (preliminarily described as “others”; expected to be of non-insect origin, e.g. bacteria) against the Genbank database, employing the basic local alignment search tool (blast)n algorithm, and the output of this query was subsequently analyzed in MEGAN6 applying the lowest common ancestor (LCA) algorithm to produce a final taxonomic assignment [33]. For hierarchical visualization of recovered taxonomic composition of mock samples we used Krona charts [34].

#### MiSeq recovery of diversity information

To separately assess the recovery capacity of metabarcoding (i.e. MiSeq procedure) we checked the overlap of MiSeq-produced sequencing clusters against the Sanger data produced from the specimens included in the mock samples. We performed blast searches of all representative sequences from all MiSeq-produced MOTU clusters against a reference library consisting of standard barcoding sequences from all specimens included in the mock samples. The minimal e-value for blast searches was set to 1e-10 and the similarity threshold was set to 97%.

#### Statistical analyses

We evaluated the success of individual methods (morphological approach, standard barcoding, and metabarcoding) for identifying host–parasitoid members of five mock samples at different taxonomic levels (family, genus, species) using the series of proportion tests [35] performed for each taxonomic level in R 3.2.2. [36].

### Standard barcoding vs morphotyping

#### Morphological identification

Hosts were morphotyped and assigned into morphospecies by appropriate specialists (as above) and parasitoids were identified by M. Šigut. These were mostly identified to higher taxonomic levels because expert-level species identifications are usually a long-term process, especially in the case of large-scale projects, and the experience of M. Šigut on the identification of these specimens is intermediate (i.e. roughly 400 h before the project plus 800 h during this project). Parasitoid specimens are currently being identified by taxonomists and accurate identifications will be continuously updated on BOLD as they become available.

#### DNA barcoding

A total of 1037 specimens (417 hosts plus 620 parasitoids) were analyzed at the Canadian Centre for DNA Barcoding (University of Guelph) using standardized protocols [37, 38]. Thirty-five parasitoids were processed at the University of Ostrava, Czech Republic (performing sequencing at Macrogen Inc., Seoul, South Korea) using the same protocols as in mock samples’ processing. Generated sequences were assigned to BINs and identified using BOLD analytical tools. All DNA sequences and respective specimens are accessible on BOLD (dataset DS-LANZMET, DOI dx.doi.org/10.5883/DS-LANZMET). Voucher specimens are deposited at the University of Ostrava and ZSM.

## Results

### Morphological, standard barcoding, and metabarcoding identifications

**Fig 1:**
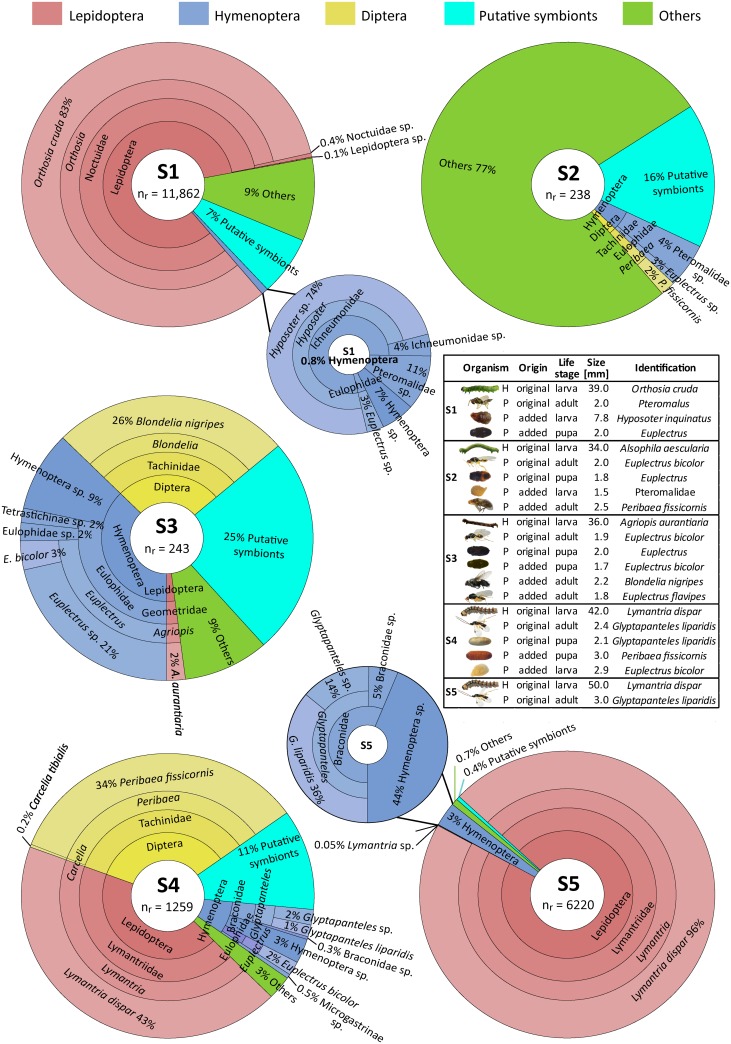
Detailed composition of the individual mock samples (S1–S5) recovered by DNA metabarcoding (Illumina MiSeq). The taxonomic assignment of recovered host and parasitoid taxa is emphasized together with the proportion of putative symbionts. The width of each sector corresponds to the relative proportion of its reads (n_r_ = total number of reads). Taxonomic levels are displayed hierarchically from order (the innermost layer) to species level (the outermost layer). The inset table shows organisms (H = host, P = parasitoid) put in the mock samples, and their identification to the lowest possible taxonomic level based on consensus of the three methods (morphological identification, standard barcoding and metabarcoding). See S1 and S3 Tables for details.

Based on the mock samples, there were no significant differences between the three methods regarding identification success into individual taxonomic levels (*χ2* = 2.03, df = 2, *P* = 0.362; *χ2* = 5.12, df = 2, *P* = 0.078; and *χ2* = 0.50, df = 2, *P* = 0.780, for family, genus and species level, respectively; Table 1, S1 Table). The metabarcoding successfully generated 19,963 forward and 20,155 reverse reads after quality trimming. On average, we retained 3992 forward and 4031 reverse reads per sample with median read length of 254 and 252 bp, respectively. After performing all quality processing and clustering procedures and discarding the generated singletons, 19,822 reads were retained (i.e. ~50% of all reads). Their clustering resulted in 69 different MOTUs (S3 Table) including eight (11.6%) host clusters (82.7% of reads; n = 16,386) and 15 (21.7%) parasitoid clusters (4.88% of reads; n = 966). Interestingly, 46 MOTUs (66.7%) had no match with standard COI barcoding reference sequences; 27 of these clusters (39.1%) were further identified as putative symbionts (5.39% of reads; n = 1068) and 19 (27.5%) were assumed as environmental or laboratory contaminants (6.63% of reads; n = 1315). Eighty-seven reads (0.44%) were not assigned to a taxonomically specific cluster and might, therefore, represent chimeric sequences (with no similar hit in the reference database and/or with various parts of the same sequence showing very different taxonomic identifications) that were not excluded by the *de novo* chimera identification procedure (Fig 1).

**Table 1:** Success of each method in the identification of the 22 individuals present in the mock samples.

Method	% Identification success		
	Family	Genus	Species
Morphological identification	100.0	59.1	54.5
Standard barcoding	100.0	86.4	63.6
Metabarcoding	95.5	81.8	54.5

Further details are indicated in S1 Table.

The overall metabarcoding diversity recovery was high, reaching 92.8% (13/14) of the host and parasitoid taxa (unique BINs), and 95.5% (21/22) of the individuals employed in this study had successful blast hits in the local reference library consisting of representative standard barcoding sequences from all specimens included in the mock samples (S1 Fig). Only one individual belonging to *Alsophila aescularia* (lepidopteran host) was not recovered and thus could not be assigned.

### Standard barcoding vs morphotyping

Standard barcode (i.e. COI) sequences were recovered from 865 of the 1072 analyzed specimens resulting in 795 (465 parasitoid, 330 host) barcode compliant records (>500 bp; 74.2%). In total, we obtained 104 different parasitoid and 108 host BINs. The 795 DNA barcodes obtained increased the putative diversity of assigned parasitoid morphospecies in our studied area from 91 to 104 BINs (MOTUs), with subfamilies Eulophinae and Microgastrinae presenting the highest increase (from six to 14, and from 19 to 26, respectively). Similarly, barcodes recovered from hosts increased their putative diversity from 97 morphospecies to 108 BINs, with families Geometridae and Tortricidae presenting the highest increase (from 29 to 34, and from eight to 12, respectively). Regarding the completeness and usability of these local reference libraries, the identification success of herbivore hosts was much higher than that of parasitoids. In hosts, we successfully identified 90.7% of BINs to the species level (98/108), whereas in parasitoids, identification success was limited to higher taxonomic levels with only 39.4% (41/104) of BINs successfully identified as putative species (Table 2).

**Table 2:** Host–parasitoid diversity at the study site (Lanžhot, Czech Republic) sampled in 2013–2014.

Taxonomic group	No. of barcoded specimens	No. of larva/pupa/adult	No. of morphospecies	No. of BINs	% of BINs assigned to:			
					Family	Subfamily	Genus	Species
**Hymenoptera**								
Agathidinae (B)	1	1/0/0	1	1	100.0	100.0	100.0	0.0
Anomaloninae (I)	1	0/0/1	1	1	100.0	100.0	100.0	100.0
Banchinae (I)^a^	5	0/0/5	5	4	100.0	50.0	50.0	25.0
Campopleginae (I)^a^	80	1/3/76	17	15	100.0	60.0	53.3	33.3
Cryptinae (I)	4	0/0/4	3	3	100.0	66.7	66.7	66.7
Entedoninae (CH)	2	0/0/2	1	1	100.0	0.0	0.0	0.0
Eulophinae (CH)^a^	104	1/3/100	6	14	92.9	64.3	64.3	14.3
Euphorinae (B)	3	0/3/0	1	1	100.0	100.0	100.0	100.0
Homolobinae (B)	1	0/0/1	1	1	100.0	100.0	0.0	0.0
Hormiinae (B)	1	0/0/1	1	1	100.0	100.0	0.0	0.0
Macrocentrinae (B)	7	0/0/7	2	2	100.0	100.0	100.0	0.0
Mesochorinae (I)^a^	13	0/1/12	6	5	100.0	40.0	20.0	20.0
Microgastrinae (B)^a^	96	0/20/76	19	26	96.2	96.2	53.8	26.9
Ophioninae (I)	8	4/3/1	3	3	66.7	66.7	66.7	66.7
Orgilinae (B)	2	0/0/2	1	1	100.0	100.0	0.0	0.0
Perilampinae (CH)	6	0/6/0	1	1	0.0	0.0	0.0	0.0
Pimplinae (I)	1	0/0/1	1	1	100.0	100.0	100.0	100.0
Pteromalinae (CH)	1	0/1/0	1	1	100.0	0.0	0.0	0.0
Rogadinae (B)^a^	16	0/2/14	1	2	100.0	100.0	100.0	100.0
Tryphoninae (I)	1	1/0/0	1	1	100.0	100.0	100.0	100.0
**Diptera**								
Dexiinae (T)	5	0/5/0	2	2	100.0	50.0	50.0	50.0
Exoristinae (T)^a^	86	1/78/7	10	12	100.0	91.7	91.7	91.7
Tachininae (T)^a^	18	0/13/5	4	3	100.0	100.0	100.0	100.0
other^b^	3	2/1/0	2	2	0.0	0.0	0.0	0.0
**∑**	**465**	**11/139/315**	**91**	**104**	**94.2**	**75.0**	**59.6**	**39.4**
Argidae (H)	1	1/0/0	1	1	100.0	100.0	100.0	100.0
Bucculatricidae (L)	10	8/0/2	3	3	100.0	100.0	100.0	66.7
Depressariidae (L)	2	2/0/0	1	1	100.0	100.0	100.0	100.0
Drepanidae (L)	2	2/0/0	1	1	100.0	100.0	100.0	100.0
Erebidae (L)	26	22/1/3	6	7	100.0	100.0	100.0	100.0
Gelechiidae (L)	6	6/0/0	6	6	100.0	100.0	83.3	83.3
Geometridae (L)	145	99/37/9	29	34	100.0	100.0	97.1	88.2
Gracillariidae (L)	4	4/0/0	3	3	100.0	100.0	100.0	66.7
Limacodidae (L)	1	0/1/0	1	1	100.0	100.0	100.0	100.0
Lypusidae (L)	20	20/0/0	3	3	100.0	100.0	100.0	66.7
Noctuidae (L)	41	33/6/2	14	13	100.0	100.0	100.0	92.3
Nolidae (L)	11	10/1/0	3	3	100.0	100.0	100.0	100.0
Notodontidae (L)	8	8/0/0	5	5	100.0	100.0	100.0	100.0
Psychidae (L)	15	11/3/1	4	6	100.0	100.0	100.0	83.3
Pyralidae (L)	1	1/0/0	1	1	100.0	100.0	100.0	100.0
Roeslerstammiidae (L)	2	2/0/0	2	2	100.0	100.0	100.0	100.0
Tenthredinidae (H)	6	5/1/0	3	3	100.0	100.0	100.0	100.0
Tortricidae (L)	26	16/4/6	8	12	100.0	100.0	100.0	100.0
Ypsolophidae (L)	3	2/0/1	3	3	100.0	100.0	100.0	100.0
**∑**	**330**	**252/54/24**	**97**	**108**	**100.0**	**100.0**	**98.1**	**90.7**

The completeness and usability of the BOLD reference database (version 4; 16. 6. 2016) is demonstrated on the identification success of host and parasitoid BINs into individual taxonomic levels (family, subfamily, genus, species) and compared to the number of morphospecies obtained.

All BINs were assigned to the Order level.

Parasitoid taxa are shaded blue, host taxa red.

B = Braconidae, I = Ichneumonidae, CH = Chalcidoidea, T = Tachinidae, H = Hymenoptera, L = Lepidoptera.

^a^Taxa with different number of BINs and morphospecies

^b^Specimens impossible to assign to any taxonomic group

## Discussion

### Metabarcoding, standard barcoding, and morphological identifications performance

Overall, recovered diversity and identification precision using metabarcoding agreed with that obtained using standard barcoding and accurately reflected morphological identifications. Thus, our data suggest that employing both paired-end reads in a parallel manner in the classification procedure used in metabarcoding produced assignments as accurate as in the standard barcoding approach (except in one case; see specimen “*Hyposoter*” in sample S1 in S1 Table). Despite this accuracy, we recorded an inferior precision (although non-significant) of metabarcoding identification when compared to standard barcoding, resulting from the overall shorter sequences produced by the MiSeq platform (658 bp vs. ~2*300 bp). This was tested *in silico* for above-mentioned specimen by cutting out the missing part from the middle of its full barcode sequence: in such case, RTAX confirmed the same taxonomic assignment as with MiSeq representative read. Nevertheless, the metabarcoding approach, using a combination of MiSeq sequencing, amplification of almost full COI barcodes by means of updated wide-coverage degenerate primers, and bioinformatic procedures including RTAX classification, seems to effectively recover host–parasitoid diversity. A similar conclusion was reached in a plant diversity study using metabarcoding, showing that full-length barcode markers have a potential to outperform shorter barcode fragments [39]. An alternative to the RTAX method for processing the classification of paired-end reads would be to search concatenated reads with N’s filling the gap of non-overlapping parts of the markers against a reference library; however, current search algorithms, as that implemented in BLAST or USEARCH, do not produce ideal results with this type of query [32] and, therefore, RTAX is the most effective solution. Compared to other currently available classifiers, RTAX seems to suffer from over classification and higher error rates, particularly at the species and genus levels, related to the coverage of the reference database [40]. However, its main indisputable advantage remains: the ability to use both non-overlapping reads producing doubled information, which has been proven to result in better classifications compared to partial information from single read and thus shorter overall sequence [39]. In our study, we used a conservative (3%) pairwise sequence divergence as the threshold to distinguish between two MOTUs, although the arbitrary nature of this clustering dissimilarity threshold has been criticized and alternative methods of MOTU delimitations are available (see [11] and references therein).

The design of degenerate primers applied in the present study might yet be suboptimal for the amplification and sequencing of particular species as we were unable to detect the host *Alsophila aescularia*. Although Sanger sequencing was able to recover the COI sequence of this lepidopteran using standard insect primers, an attempt to generate Sanger sequences using Fol-degen primers repeatedly failed. The unsuitability of degenerate primers for *Alsophila* species might be further confirmed when the mitochondrial genome of any of the *Alsophila* species becomes available. Another inconvenience of using highly degenerated primers is their sensitivity to environmental or laboratory contaminations [41]. This aspect was confirmed by our study as our metabarcoding data contained obvious contaminants such as plant (e.g. Malphigiales, *Solanum lycopersicum*), leaf beetle (family Chrysomelidae, SPH01), or endemic Australian butterfly *Leucania cruegeri* DNA (S3 Table). In our particular case, laboratory contaminations might have had a greater impact than that expected for the practical application of metabarcoding due to the excessive manipulation of specimens during the rearing, preparation of artificial mock samples, and morphological identification procedures. Rinsing and carefully handling specimens during processing could eliminate a substantial part of environmental and laboratory contaminations.

Another inconvenience of using metabarcoding is the need for a careful interpretation of the recovered diversity as more insect species can be revealed than that involved in the real interaction. In multiple hosts’ detection, this situation is simply resolved because the DNA of the real host is usually present in the largest amount. However, in multiple parasitoids’ detection, deciding which species was indeed present in the sample is much more complicated as one host can be parasitized by multiple species, and the amount of their DNA in the sample is usually an order of magnitude lower than that of their host. In our particular case, in addition to revealing the identity of the parasitoids added to the mock samples, DNA metabarcoding revealed parasitoid taxa of uncertain origin: *Carcelia tibialis* (0.2%, sample S4), *Microplitis demolitor* (0.03%, sample S5), and a Tetrastichinae species (2%, sample S3). Since *M*. *demolitor* is a non-European species commonly used in insect pest management, and its DNA was present in a very small amount, this was considered a laboratory contamination. However, *C*. *tibialis* is common in Europe, *Carcelia* spp. are known to attack the host in which *C*. *tibialis* was detected, and it was represented in a considerable amount of reads; thus, this species might indeed have been present in the host's body but it was overlooked during dissection. Similarly, Tetrastichinae parasitoids are known to attack the host where they were found, and due to the relatively high number of reads we can assume that representatives of this subfamily were present in the samples. Alternatively, they might have been hyperparasitoids of added or dissected parasitoid species [42]. Moreover, parasitoids’ oviposition in the ‘wrong’ host is common [43] and, therefore, detected parasitoid DNA could have originated from such unsuccessful parasitation attempts. However, unpredicted taxa in our samples could as well represent misclassifications based on the misidentified hits in reference database, which can occur more frequently in understudied and taxonomically challenging groups [44]. These aspects emphasize the importance of building accurate and comprehensive reference libraries and knowing the ecology of detected insect species.

Standard DNA barcoding is not affected by most of the above-mentioned issues arising from the nature of mixed samples. However, a common problem of Sanger sequencing in the identification of host–parasitoid interactions are the cross contaminations between host and parasitoid DNA [13]. Metabarcoding not only eliminates such problems, as it can also use them to its own benefit by simultaneously detecting and identifying host and parasitoids without rearing or dissecting procedures, which are common time-consuming and labor intensive procedure linked to the classical morphological approach [13]. Moreover, the morphological identification of host–parasitoid communities requires experienced taxonomists as these are composed of larval and/or other hardly identifiable developmental stages. Cryptic species, common in many parasitoid taxa [12, 14, 45], and unsuccessfully reared developmental stages often represent a substantial proportion of dataset (see Table 2 for example) and further complicate their identification. Molecular methods allow detecting such stages and cryptic species and, by increasing the recovered host–parasitoid diversity, can substantially extend the final dataset. This aspect was confirmed in our study as the morphological approach failed to classify a substantial part of the parasitoid samples to the genus and species levels (eight unclassified individuals in larval and pupal stages), while these samples were identified by both molecular methods. However, molecular methods are unable to distinguish special life strategies (e.g. hyperparasitism) and to prove successful parasitism [46]. For these situations, rearing methods allowing the observation of all life stages and the outcome of such interactions seem to be more appropriate. Moreover, relying exclusively on the molecular methods for the species description is inadequate, mainly because building species hypothesis based on a single locus is inappropriate [47]. Still, combining sequence data and other evidence is becoming a widely accepted practice [48, 49].

A considerable part of the diversity of our mock samples recovered by metabarcoding was composed of putative endosymbionts (5.39% of all reads and 27 taxonomic clusters; Fig 1, S3 Table) suggesting its potential for simultaneously recovering endosymbiotic microbiota. It is known that endosymbionts may shape the structure and dynamics of insect food webs [50, 51], and examining insect hosts and their associated microbiota has been tried or discussed [18]. However, we were unable to confirm the endosymbiotic origin of most taxa as many of them were facultative endosymbionts (e.g. can be found either as epiphytic or gut microflora [51–53]); this was the case for *Pseudomonas*, *Stenotrophomonas*, *Acinetobacter*, or *Acetivibrio*. Conversely, we assumed the endosymbiotic origin of *Rickettsia*, *Ralstonia*, or *Kocuria*, which are obligate endosymbiotic genera. In addition, many endosymbionts identified in our study were not host-specific; *Rickettsia*, for example, is known from the gut microflora of various herbivorous insects but also from the eulophid wasp, *Pnigalio soemius* [54, 55]. On the other hand, the cellulolytic bacteria *Ralstonia* were probably associated with lepidopteran hosts instead of parasitoids (e.g. [55]). Moreover, most endosymbionts’ clusters were only identified to high taxonomic levels (S3 Table) and a better recognition of their origin requires more accurate taxonomic identifications, which could be improved by using more specific bacterial and fungal primers with growing reference libraries and knowledge on endosymbiont ecology.

### Standard barcoding vs morphotyping

The second part of our study compared the accuracy of molecular (DNA barcoding) and morphological approaches for the potential reconstruction of a local food web, and assessed the completeness of a local reference library. The previously discussed benefits of DNA barcoding in comparison with morphological identification were evidenced in the markedly increased parasitoid diversity, especially in Eulophinae and Microgastrinae. This agrees with previous studies confirming the high level of crypsis and identification issues in both these groups [12, 56]. Similarly, in hosts, the highest increase in diversity was obtained for Geometridae and Tortricidae, probably due to the presence of hardly identifiable juveniles, and to the high level of crypsis of both groups, which is well documented in the literature [57, 58].

In the Czech Republic, the focal community of lepidopteran and hymenopteran hosts and their parasitoids includes approx. 4000 host and 4500 parasitoid species [59–61]. The reference library of hosts is quite extensive and, from our dataset, we successfully identified the vast majority of putative species (BINs) to the species level while the identification success of parasitoids to the species level was much lower (Table 2). These identification success rates are similar to those reported for the tropics, where 87% of the host and 36% of the parasitoid sequences were successfully identified to the species level based on Papua New Guinea’s local reference library [13]. Successful molecular identifications of central European hosts and their parasitoids benefit strongly from the growing number of large-scale barcoding campaigns, such as the Barcoding Fauna Bavarica [62] or the German Barcoding of Life commenced in 2012, which are substantially contributing to build reference libraries. However, the low efficiency of parasitoids’ molecular identification compared to that of their hosts is caused by their huge unrevealed diversity and lack of molecular data [2] and by taxonomic issues [13]. This seems to be particularly evident in some hyperdiverse and/or cryptic groups of hymenopteran parasitoids, which showed the lowest number of BINs assigned to species (e.g. Eulophinae, 14.3%; Microgastrinae, 26.9%; Campopleginae, 33.3%; Table 2). The situation is much better in European tachinids (Diptera), with 88.2% of BINs identified to species, suggesting that they represent a well-studied and well-barcoded group. These results emphasize the need to continue building comprehensive reference libraries focused on parasitoids.

### Future of metabarcoding identifications

Our study proposes using metabarcoding for effectively recovering host–parasitoid diversity. As indicated above, this approach will be most effective at localities where species diversity is covered by comprehensive reference libraries, which is still far from reality, especially for parasitoids. Moreover, accurate identifications are still needed. From the methodological point of view, we suggest that a further increase in recovered diversity could stem from the use of multiple independent markers, which are being increasingly developed as reference databases for alternative barcoding markers also increase [63–66]. In terms of classification procedures, a wide array of algorithms and methods has been developed for this purpose, but none seems to be ideal for all eventualities [63]. Recently, a statistical algorithm capable of incorporating data from several independent classifiers as covariates in a probabilistic taxon membership assignment process was developed, providing unbiased probabilities of taxonomic placement [67]. Thus, there is a growing number of tools helping with this aspect and providing as precise and accurate classifications as possible.

To efficiently process a multitude of samples in high-throughput mode and still be able to draw significant conclusions and capture sparse interactions, several alternative approaches were designed. One of them, multiplex PCR, was suggested to decrease analyses’ costs, allowing the simultaneous detection of several target species, and, in combination with two-dimensional sample pooling, decreasing the number of PCR reactions up to 90% [68]. This will be particularly useful for the specific screening the limited number of interactions with low parasitism rates (<10%), while metabarcoding is more useful for the more general detection of all present trophic links involving unexpected interactions. Multiparasitism or hyperparasitism are quite common [69], but, in general, only a few parasitoid species share one host. Recent advances in sample multiplexing allowed new indexing schemes, including double-indexing or nested tagging [6], and the expected diversity recovered per sample and read depth needed increased with the continuously increasing output of modern sequencers (e.g. Illumina NovaSeq series); thus, a large number of samples can now be pooled and should easily cover the needs of any ecological study. Based on our results, one MiSeq run could potentially be used to process thousands of samples (~5000).

The primary aim of our study was to test metabarcoding as an alternative to standard barcoding and classical morphological identifications, both time-consuming and labor intensive, for a prospective large-scale study of host–parasitoid interactions. Due to limited number of mock samples used in our study, further testing, including other host and parasitoid taxa to cover more of the real global diversity, would be necessary to draw more general and robust conclusions. Still, the present study might be a starting point for further comparative studies. Although in our study, the performance of metabarcoding was tested on mock samples, each including one host and multiple parasitoids (except of S5), for the purpose of studies of host–parasitoid interactions, each host larva potentially with parasitoid(s) inside would be a separately barcoded inclusion in the sequencing pool. Many different aspects of HTS are known to bring technical issues into the datasets produced by these techniques, including library preparation biases, stochastic effects during sequencing, and bioinformatic analyses biases. These are being extensively discussed and subjected to thorough testing in relevant stand-alone studies, and recent findings have shown that those negative effects could be effectively solved by simply increasing sequencing depth [70]. However, optimal sequencing depth for recovery of full complexity of the sample involving host with all (potential) parasitoids inside is tricky to define as it depends on an array of conditions. The most relevant are size proportions of organisms involved in the sample and amplification efficiency. Nevertheless, given that high-throughput costs are continuing to decline, we believe that with growing accurate and comprehensive reference libraries and increasing knowledge on the ecology of detected insect species, metabarcoding might be the most effective future solution for large-scale studies of host–parasitoid food webs, allowing the simultaneous assessment of associated microbiota, thus saving both time and money. However, molecular methods cannot entirely replace the reconstruction of ecological interactions by direct observation (e.g. rearing), and, for their accurate interpretation, reliable taxonomic backgrounds will still be needed.

## Supporting information

S1 Table Taxonomic assignments of hosts and parasitoids present in mock samples by morphological identification, standard barcoding, and metabarcoding. (PDF)

S2 Table List of taxonomic resources used for morphological identification of parasitoids present in mock samples. (PDF)

S3 Table Overview of clusters (MOTUs) recovered by MiSeq from individual mock samples. (PDF)

S1 Fig Phylogenetic reconstruction of representative sequences of all MOTUs generated from MiSeq together with standard barcode sequences from all hosts and parasitoids present in mock samples. (PDF)
