# Supplementary material for: Performance of DNA metabarcoding, standard barcoding, and morphological approach in the identification of host–parasitoid interactions
Source: PLoS One. 2017 Dec 13;12(12):e0187803. doi: 10.1371/journal.pone.0187803 (PMC5728528; doi:10.1371/journal.pone.0187803)
Supplement: S1 Table — (PDF) [file pone.0187803.s001.pdf]

S1 Table. Taxonomic assignments of hosts and parasitoids present in mock samples by morphological identification, standard barcoding, and metabarcoding.

Sample	Organism	Origin	Life stage	Size (mm)	BIN	Mopholological identification	Standard barcoding	Metabarcoding
S1	host	original	larva	39.0	AAD9024	*Orthosia cruda*	*Orthosia cruda*	*Orthosia cruda*
	parasitoid	original	adult	2.0	ACJ7542	*Pteromalus*	Pteromalidae	Pteromalidae
	parasitoid	added	larva	7.8	AAU8361	Ichneumonidae	*Hyposoter inquinatus*	*Hyposoter*
	parasitoid	added	pupa	2.0	ACU3595	Eulophidae	*Euplectrus*	*Euplectrus*
S2	host	original	larva	34.0	AAC9096	*Alsophila aescularia*	*Alsophila aescularia*	unidentified
	parasitoid	original	adult	2.0	ACU3753	*Euplectrus bicolor*	*Euplectrus*	*Euplectrus*
	parasitoid	original	pupa	1.8	ACU3753	Eulophidae	*Euplectrus*	*Euplectrus*
	parasitoid	added	larva	1.5	ACJ7542	Pteromalidae	Pteromalidae	Pteromalidae
	parasitoid	added	adult	2.5	ACH1961	*Peribaea fissicornis*	*Peribaea fissicornis*	*Peribaea fissicornis*
S3	host	original	larva	36.0	AAC2753	*Agriopis aurantiaria*	*Agriopis aurantiaria*	*Agriopis aurantiaria*
	parasitoid	original	adult	1.9	ACU3230	*Euplectrus bicolor*	*Euplectrus*	*Euplectrus*
	parasitoid	original	pupa	2.0	ACU3230	Eulophidae	*Euplectrus*	*Euplectrus*
	parasitoid	added	pupa	1.7	ACR7505	Eulophidae	*Euplectrus bicolor*	*Euplectrus bicolor*
	parasitoid	added	adult	2.2	AAN4077	*Blondelia nigripes*	*Blondelia nigripes*	*Blondelia nigripes*
	parasitoid	added	adult	1.8	ACU2970	*Euplectrus flavipes*	Eulophidae	Eulophidae
S4	host	original	larva	42.0	AAA2052	*Lymantria dispar*	*Lymantria dispar*	*Lymantria dispar*
	parasitoid	original	adult	2.4	AAV2164	*Glyptapanteles liparidis*	*Glyptapanteles liparidis*	*Glyptapanteles liparidis*
	parasitoid	original	pupa	2.1	AAV2164	Braconidae	*Glyptapanteles liparidis*	*Glyptapanteles liparidis*
	parasitoid	added	pupa	3.0	ACH1961	Tachinidae	*Peribaea fissicornis*	*Peribaea fissicornis*
	parasitoid	added	larva	2.9	ACR7505	Eulophidae	*Euplectrus bicolor*	*Euplectrus bicolor*
S5	host	original	larva	50.0	AAA2052	*Lymantria dispar*	*Lymantria dispar*	*Lymantria dispar*
	parasitoid	original	adult	3.0	AAV2164	*Glyptapanteles liparidis*	*Glyptapanteles liparidis*	*Glyptapanteles liparidis*

Altogether, 17 parasitoid and 5 host specimens representing 14 putative species (BINs) were analyzed.
