# Supplementary material for: Performance of DNA metabarcoding, standard barcoding, and morphological approach in the identification of host–parasitoid interactions
Source: PLoS One. 2017 Dec 13;12(12):e0187803. doi: 10.1371/journal.pone.0187803 (PMC5728528; doi:10.1371/journal.pone.0187803)
Supplement: S2 Table — (PDF) [file pone.0187803.s002.pdf]

S2 Table. List of taxonomic resources used for morphological identification of parasitoids present in mock samples.

Parasitoids in general

Goulet H, Huber JT. Hymenoptera of the world: an identification guide to families. Ottawa: Research Branch, Agriculture Canada; 1993.

Šedivý J. Checklist of Czechoslovak Insects III (Hymenoptera). Acta Entomol Mus Natl Pragae. 1989; 19: 1–194.

Ichneumonoidea

Austin AD, Dangerfield PC. Synopsis of Australasian Microgastrinae (Hymenoptera: Braconidae), with a key to genera and description of new taxa. Invertebr Taxon. 1992; 6: 1–76.

Broad G. Identification key to the subfamilies of Ichneumonidae (Hymenoptera). London: The Natural History Museum; 2011.

Horstmann K. Bemerkungen zur Systematik einiger Gattungen der Campopleginae (Hymenoptera, Ichneumonidae). Nachrichtenbl Bayer Entomol. 1970; 19: 77–84.

Horstmann K. Bemerkungen zur Systematik einiger Gattungen der Campopleginae II (Hymenoptera, Ichneumonidae). Mitt Münch Entomol Ges. 1977; 67: 65–83.

Horstmann K. Bemerkungen zur Systematik einiger Gattungen der Campopleginae III (Hymenoptera, Ichneumonidae). Mitt Münch Entomol Ges. 1986; 76: 143–164.

Horstmann K. Bemerkungen zur Systematik einiger Gattungen der Campopleginae IV (Hymenoptera, Ichneumonidae). Z Arbeitsgem Osterr Entomol. 2004; 56: 13–35.

Perkins JF. Ichneumonidae, key to subfamilies and Ichneumoninae – 1. Handbk Ident Br Insects. 1959; 7: 1–116.

Shaw MR, Huddleston T. Classification and biology of braconid wasps (Hymenoptera: Braconidae). Handbk Ident Br Insects. 1991; 7: 1–126.

Van Achterberg C. Illustrated key to the subfamilies of the Holarctic Braconidae (Hymenoptera: Ichneumonoidea). Zool Meded. 1990; 64: 1–20.

Van Achtenberg C. Fauna Europaea – Ichneumonoidea. 2013. Available from: http://faunaeur.org.

Venkatesha MG, Gopinath K. Description of immature stages of a species of *Glyptapanteles* (Hymenoptera: Braconidae), a gregarious endoparasitoid of *Amata passalis* (Fabricius) (Lepidoptera: Arctiidae), a defoliator of sandalwood, *Santalum album* L. Insect Sci Appl. 1994; 15: 161–165.

Yu DS, van Achterberg C, Horstmann K. Home of Ichneumonoidea. 2012. Available from: http://www.taxapad.com.

Chalcidoidea

Bouček Z, Rasplus J-Y. Illustrated key to west-Palearctic genera of Pteromalidae (Hymenoptera: Chalcidoidea). Paris: Institut National de la Recherche Agronomique; 1991.

Dzhanokmen KA. A review of pteromalids of the genus *Pteromalus* Swederus (Hymenoptera, Pteromalidae) of Kazakhstan. II. Entomol Obozr. 2001; 80: 472–496.

Gibson GAP, Huber JT, Woolley JB. Annotated keys to the genera of Nearctic Chalcidoidea (Hymenoptera). Ottawa: NRC Research Press; 1997.

Graham MWRDV. The Pteromalidae of north-western Europe (Hymenoptera: Chalcidoidea). Bull Br Mus (Nat Hist) Entomol. 1969; Suppl 16: S1–S908.

Hansson C, Smith MA, Janzen DH, Hallwachs W. Integrative taxonomy of New World *Euplectrus* Westwood (Hymenoptera, Eulophidae), with focus on 55 new species from Area de Conservación Guanacaste, northwestern Costa Rica. Zookeys. 2015; 485: 1–236.

Kalina V. Checklist of Czechoslovak Insects III (Hymenoptera). Chalcidoidea. Acta Entomol Mus Natl Pragae. 1989; 19: 97–127.

Noyes JS. Universal Chalcidoidea Database. 2016. Available from: http://nhm.ac.uk/chalcidoids.

Peck O, Bouček Z, Hoffer A. Keys to the Chalcidoidea of Czechoslovakia (Insecta: Hymenoptera). Mem Entomol Soc Can. 1964; 96: 7–121.

Schauff ME, Janzen DH. Taxonomy and ecology of Costa Rican *Euplectrus* (Hymenoptera: Eulophidae), parasitoids of caterpillars. J Hymenoptera Res. 2001; 10: 181–230.

Sureshan PM. Studies on *Pteromalus* Swederus (Hymenoptera: Chalcidoidea: Pteromalidae) of the Indian subcontinent with the description of three new species. Rec Zool Surv India. 2001; 99: 5–14.

Zhu C-D, Huang D-W. A study of the genus *Euplectrus* Westwood (Hymenoptera : Eulophidae) in China. Zool Stud. 2003; 42: 140–164.

Tachinidae

Herting B. Catalogue of Palearctic Tachinidae (Diptera). Stuttg Beitr Naturkd (A Biol). 1984; 369: 1–228.

Chvála M. Check list of Diptera (Insecta) of the Czech and Slovak Republics. Prague, Czech Republic: Karolinum, Charles University Press; 1997.

Malloch JR. A preliminary classification of Diptera, exclusive of Pupipara, based upon larval and pupal characters, with keys to imagines in certain families. Ill Nat Hist Surv Bull. 1917; 12: 161–409.

Sabrosky CW, Reardon RC. Tachinid parasites of the gypsy moth, *Lymantria dispar*, with keys to adults and puparia. Misc Publ Entomol Soc Am. 1976; 10: 1–126.

Tschorsnig H-P, Herting B. The tachinids (Diptera: Tachinidae) of Central Europe: identification keys for the species and data on distribution and ecology. Stuttg Beitr Naturkd (A Biol). 1994; 506: 1–170.

Vaňhara J, Tschorsnig H-P, Herting B, Mückstein P, Michalková V. Annotated host catalogue for the Tachinidae (Diptera) of the Czech Republic. Entomol Fenn. 2009; 20: 22–48.
