# Supplementary material for: Performance of DNA metabarcoding, standard barcoding, and morphological approach in the identification of host–parasitoid interactions
Source: PLoS One. 2017 Dec 13;12(12):e0187803. doi: 10.1371/journal.pone.0187803 (PMC5728528; doi:10.1371/journal.pone.0187803)
Supplement: S3 Table — (PDF) [file pone.0187803.s003.pdf]

S3 Table. Overview of clusters (MOTUs) recovered by MiSeq from individual mock samples.

Cluster	S1		S2		S3		S4		S5		Total		Origin
	No	%	No	%	No	%	No	%	No	%	No	%	
Acetivibrio	2	0.02	0	0.00	0	0.00	0	0.00	0	0.00	2	0.01	PS
Acinetobacter	2	0.02	0	0.00	0	0.00	0	0.00	0	0.00	2	0.01	PS
Agriopis aurantiaria	0	0.00	0	0.00	5	2.06	0	0.00	0	0.00	5	0.03	HOST
Alphaproteobacteria	3	0.03	0	0.00	0	0.00	0	0.00	0	0.00	3	0.02	PS
Amyelois transitella	3	0.03	0	0.00	0	0.00	0	0.00	0	0.00	3	0.02	O
Athetis nonagrica	3	0.03	0	0.00	0	0.00	0	0.00	0	0.00	3	0.02	O
Bacilli	0	0.00	0	0.00	0	0.00	2	0.16	3	0.05	5	0.03	PS
Bacteria	2	0.02	0	0.00	0	0.00	0	0.00	0	0.00	2	0.01	PS
Bacteroidales	2	0.02	0	0.00	0	0.00	5	0.40	2	0.03	9	0.05	PS
Bacteroides	0	0.00	0	0.00	0	0.00	2	0.16	0	0.00	2	0.01	PS
Bifidobacterium	3	0.03	2	0.84	0	0.00	0	0.00	0	0.00	5	0.03	O
Bilateria	2	0.02	0	0.00	0	0.00	0	0.00	0	0.00	2	0.01	O
Blondelia nigripes	0	0.00	0	0.00	64	26.34	0	0.00	0	0.00	64	0.32	PAR
Bombyx mori	3	0.03	0	0.00	0	0.00	2	0.16	0	0.00	5	0.03	O
Braconidae	0	0.00	0	0.00	0	0.00	4	0.32	8	0.13	12	0.06	PAR
Bradyrhizobiaceae	4	0.03	0	0.00	0	0.00	5	0.40	3	0.05	12	0.06	PS
Burkholderiaceae	4	0.03	0	0.00	0	0.00	3	0.24	5	0.08	12	0.06	O
Burkholderiales	0	0.00	0	0.00	2	0.82	0	0.00	4	0.06	6	0.03	O
Carcelia tibialis	0	0.00	0	0.00	0	0.00	3	0.24	0	0.00	3	0.02	PAR
cellular organisms	245	2.07	177	74.37	4	1.65	18	1.43	11	0.18	455	2.30	O
Clostridiales	2	0.02	2	0.84	4	1.65	0	0.00	2	0.03	10	0.05	PS
Ditrysia (Lepidoptera)	7	0.06	0	0.00	0	0.00	0	0.00	0	0.00	7	0.04	HOST
Dothideomycetes	2	0.02	0	0.00	0	0.00	0	0.00	0	0.00	2	0.01	PS
Eukaryota	5	0.04	2	0.84	0	0.00	3	0.24	4	0.06	14	0.07	O
Eulophidae	0	0.00	0	0.00	5	2.06	0	0.00	0	0.00	5	0.03	PAR
Euplectrus	3	0.03	2	0.84	52	21.40	0	0.00	0	0.00	57	0.29	PAR
Euplectrus bicolor	0	0.00	0	0.00	8	3.29	20	1.59	0	0.00	28	0.14	PAR
Glyptapanteles	0	0.00	0	0.00	0	0.00	22	1.75	24	0.39	46	0.23	PAR
Glyptapanteles liparidis	0	0.00	0	0.00	0	0.00	17	1.35	61	0.98	78	0.39	PAR
Hadeninae	7	0.06	0	0.00	0	0.00	0	0.00	0	0.00	7	0.04	O
Helicoverpa armigera	8	0.07	0	0.00	0	0.00	0	0.00	0	0.00	8	0.04	O
Hymenoptera	6	0.05	0	0.00	22	9.05	35	2.78	74	1.19	137	0.69	PAR
Hyposoter	67	0.56	0	0.00	0	0.00	0	0.00	0	0.00	67	0.34	PAR
Chrysomelidae sp. SPH01	3	0.03	0	0.00	0	0.00	0	0.00	0	0.00	3	0.02	O
Ichneumonidae	4	0.03	0	0.00	0	0.00	0	0.00	0	0.00	4	0.02	PAR
Kineosporiaceae	2	0.02	0	0.00	0	0.00	0	0.00	0	0.00	2	0.01	PS
Kocuria	0	0.00	0	0.00	0	0.00	0	0.00	2	0.03	2	0.01	PS
Lactobacillus	4	0.03	0	0.00	0	0.00	3	0.24	4	0.06	11	0.06	PS
Lepidoptera	2	0.02	0	0.00	0	0.00	0	0.00	0	0.00	2	0.01	HOST
Leucania cruegeri	2	0.02	0	0.00	0	0.00	0	0.00	0	0.00	2	0.01	O
Lymantria	0	0.00	0	0.00	0	0.00	0	0.00	4	0.06	4	0.02	HOST
Lymantria dispar	0	0.00	0	0.00	0	0.00	543	43.13	5972	96.01	6515	32.87	HOST
Magnoliophyta	0	0.00	0	0.00	0	0.00	2	0.16	0	0.00	2	0.01	O
Malpighiales	5	0.04	0	0.00	0	0.00	0	0.00	2	0.03	7	0.04	O
Methylobacterium	0	0.00	2	0.84	0	0.00	0	0.00	0	0.00	2	0.01	PS
Microbacteriaceae	0	0.00	3	1.26	0	0.00	0	0.00	0	0.00	3	0.02	PS
Micrococcales	0	0.00	3	1.26	0	0.00	0	0.00	0	0.00	3	0.02	PS
Microgastrinae	0	0.00	0	0.00	0	0.00	6	0.48	0	0.00	6	0.03	PAR
Microplitis demolitor	0	0.00	0	0.00	0	0.00	0	0.00	2	0.03	2	0.01	O
Noctuidae	51	0.43	0	0.00	0	0.00	0	0.00	0	0.00	51	0.26	HOST
Obtectomera (Lepidoptera)	6	0.05	0	0.00	0	0.00	0	0.00	3	0.05	9	0.05	HOST
Odontodes seranensis	768	6.47	0	0.00	0	0.00	0	0.00	0	0.00	768	3.87	O
Orthosia cruda	9793	82.56	0	0.00	0	0.00	0	0.00	0	0.00	9793	49.40	HOST
Pedobacter	3	0.03	0	0.00	0	0.00	2	0.16	3	0.05	8	0.04	PS
Peribaea fissicornis	0	0.00	4	1.68	0	0.00	431	34.23	0	0.00	435	2.19	PAR
Proteobacteria	4	0.03	0	0.00	0	0.00	8	0.64	2	0.03	14	0.07	PS
Pseudomonadaceae	620	5.23	0	0.00	0	0.00	0	0.00	0	0.00	620	3.13	PS
Pseudomonas	138	1.16	21	8.82	14	5.76	76	6.04	2	0.03	251	1.27	PS
Pseudomonas fluorescens	0	0.00	0	0.00	0	0.00	3	0.24	0	0.00	3	0.02	PS
Pteromalidae	10	0.08	10	4.20	0	0.00	0	0.00	0	0.00	20	0.10	PAR
Ralstonia	0	0.00	0	0.00	0	0.00	0	0.00	2	0.03	2	0.01	PS
Rickettsia	0	0.00	0	0.00	42	17.28	0	0.00	0	0.00	42	0.21	PS
Solanum lycopersicum	8	0.07	0	0.00	0	0.00	0	0.00	0	0.00	8	0.04	O
Sphingomonadaceae	7	0.06	0	0.00	0	0.00	10	0.79	3	0.05	20	0.10	PS
Spodoptera frugiperda	3	0.03	0	0.00	0	0.00	0	0.00	0	0.00	3	0.02	O
Staphylococcaceae	0	0.00	0	0.00	0	0.00	2	0.16	0	0.00	2	0.01	PS
Stenotrophomonas rhizophila	0	0.00	0	0.00	0	0.00	26	2.07	0	0.00	26	0.13	PS
Tetrastichinae	0	0.00	0	0.00	4	1.65	0	0.00	0	0.00	4	0.02	PAR

Xanthomonadaceae	0	0.00	8	3.36	0	0.00	0	0.00	0	0.00	8	0.04	PS
Not assigned	44	0.37	2	0.84	17	7.00	6	0.48	18	0.29	87	0.44	O
∑	11862	100.00	238	100.00	243	100.00	1259	100.00	6220	100.00	19822	100.00	

Presented are MOTUs after all quality processing steps and clustering procedure with subsequent discarding of singletons. The information about number and % of reads within individual clusters and their probable origin is shown.

PAR = putative parasitoids, HOST = putative hosts, PS = putative symbionts, O = others (other taxa and not assigned sequences).
