# Supplementary material for: Performance of DNA metabarcoding, standard barcoding, and morphological approach in the identification of host–parasitoid interactions
Source: PLoS One. 2017 Dec 13;12(12):e0187803. doi: 10.1371/journal.pone.0187803 (PMC5728528; doi:10.1371/journal.pone.0187803)
Supplement: S1 Fig — (PDF) [file pone.0187803.s004.pdf]

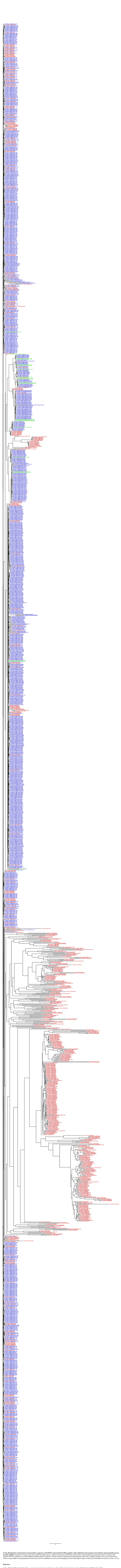

S1 Fig. Phylogenetic reconstruction of representative sequences of all MOTUs generated from MiSeq together with standard barcode sequences from all hosts and parasitoids present in mock samples. Terminal taxa in green represent standard barcoding reference sequences from mock community members, blue represent Miseq-generated representative sequences that matched with 97% similarity any of the standard barcoding reference sequences, whereas red did not match any reference database record with a required similarity. Tree was reconstructed in Fasttree using Neighbor-Joining algorithm with heuristic search for candidate joins and local bootstrapping [1] from mafft aligned sequence dataset [2] and displayed in FigTree version 1.4.2 [3].

References

1. Price MN, Dehal PS, Arkin AP. FastTree: computing large minimum evolution trees with profiles instead of a distance matrix. Mol Biol Evol. 2009; 26: 1641–1650.

2. Katoh K, Standley DM. MAFFT multiple sequence alignment software version 7: improvements in performance and usability. Mol Biol Evol. 2013; 30: 772–780.

3. Rambaut A. FigTree v1.4.2: Tree figure drawing tool. 2014. Available from: http://tree.bio.ed.ac.uk/software/figtree/.
